# Genetics and Management of the Patient with Orofacial Cleft

**DOI:** 10.1155/2012/782821

**Published:** 2012-11-01

**Authors:** Luciano Abreu Brito, Joanna Goes Castro Meira, Gerson Shigeru Kobayashi, Maria Rita Passos-Bueno

**Affiliations:** Human Genome Research Center, Institute of Biosciences, University of São Paulo, 05508-090 São Paulo, SP, Brazil

## Abstract

Cleft lip or palate (CL/P) is a common facial defect present in 1 : 700 live births and results in substantial burden to patients. There are more than 500 CL/P syndromes described, the causes of which may be single-gene mutations, chromosomopathies, and exposure to teratogens. Part of the most prevalent syndromic CL/P has known etiology. Nonsyndromic CL/P, on the other hand, is a complex disorder, whose etiology is still poorly understood. Recent genome-wide association studies have contributed to the elucidation of the genetic causes, by raising reproducible susceptibility genetic variants; their etiopathogenic roles, however, are difficult to predict, as in the case of the chromosomal region 8q24, the most corroborated locus predisposing to nonsyndromic CL/P. Knowing the genetic causes of CL/P will directly impact the genetic counseling, by estimating precise recurrence risks, and the patient management, since the patient, followup may be partially influenced by their genetic background. This paper focuses on the genetic causes of important syndromic CL/P forms (van der Woude syndrome, 22q11 deletion syndrome, and Robin sequence-associated syndromes) and depicts the recent findings in nonsyndromic CL/P research, addressing issues in the conduct of the geneticist.

## 1. Introduction

Cleft lip or palate (CL/P) is a common human congenital defect promptly recognized at birth. Despite the variability driven by socioeconomic status and ethnic background,the worldwide prevalence of CL/P is often cited as 1 : 700 live births; nevertheless, the different methods of ascertainment may lead to fluctuations in the prevalence rates [1]. Essentially, CL/P results from failure of fusion of the maxillary processes or palatal shelves, which occur between the 4th and 12th weeks of embryogenesis (as reviewed by Mossey et al. [2]). Cellular processes of proliferation, differentiation, and apoptosis, which are essential for appropriate lip and palate morphogenesis, are regulated by complex molecular signaling pathways; therefore, genetic and environmental factors that dysregulate those pathways are subject of intensive research as it is expected that their understanding will accelerate the development of preventive measures. Maternal alcohol intake or exposure to tobacco and several chemicals, such as retinoic acid and folate antagonists (e.g., valproic acid), among others, has been shown to be teratogenic, thus representing risk factors to embryos during the first trimester of pregnancy (reviewed by Bender [3] and by Dixon et al. [4]). Despite their etiological importance as environmental predisposition factors to CL/P, we will focus in this paper on the genetic causes of CL/P.

Within CL/P, cleft lip with or without cleft palate (CL ± P) is considered a distinct entity from cleft palate only (CP), based on the different embryonic origin when palate development occurs, that is, the closure of the palatal shelves occurs between 8th and 12th weeks of the human gestation [5] while lip formation is concluded at the 7th week [6]. Accordingly, this subdivision is clearly supported by epidemiological findings [4]; however, in some syndromic forms of CL/P, both entities may segregate in the same family [7–10]. CL/P can occur as the only malformation (nonsyndromic (NS), representing 70% of CL ± P cases and 50% of CP cases) or associated with other clinical features (syndromic, 30% of CL ± P and 50% of CP cases; [11]), a classification that we will consider in the next topics.

The majority of children affected by CL/P require a lasting and costly multidisciplinary treatment for complete rehabilitation. The precise clinical diagnosis of CL/P patients, which is not always simple, is crucial for an accurate genetic counseling, patient management, and definition of surgical strategies, as reviewed below. 

## 2. Genetic Factors 

### 2.1. Syndromic CL/P

Mutations in single genes and chromosomal abnormalities are the most common mechanisms underlying syndromic CL/P. The Online Mendelian Inheritance in Man database (OMIM) describes more than 500 syndromes with CL/P as part of the phenotype. Furthermore, several cases of trisomy of chromosomes 13, 18, and 21 associated with CL/P were described, as well as partial deletions and duplications of other chromosomes [12]. These findings suggest that there may be several genomic regions containing loci which, in excess or in insufficiency, may lead to CL/P.

In this paper, we highlight van der Woude syndrome (VWS) and Velocardiofacial syndrome (VCFS), due to their high frequency among CL/P cases, together with Robin sequence (RS), a clinical feature that may be associated with other syndromes, including VCFS.

#### 2.1.1. Van der Woude Syndrome (VWS)

Van der Woude syndrome (VWS; OMIM 119300), the most frequent form of syndromic CL/P, accounts for 2% of all CL/P cases [13]. VWS is a single gene disorder with an autosomal dominant pattern of inheritance. Its penetrance is high (89–99%; [14]) and it is clinically characterized mainly by CL ± P or CP, fistulae on the lower lip, and hypodontia [15]. There is a wide spectrum of clinical variability, in which patients lacking fistulae are indistinguishable from individuals affected by nonsyndromic forms. Kondo et al. [16] showed that missense and nonsense mutations in interferon regulatory factor 6 (IRF6) were responsible for the majority of VWS cases. Although the pathogenic mutations may occur in any region of the gene, about 80% of them have been found in exons 3, 4, 7, and 9 (reviewed by Durda et al. [17]). It is predicted that the pathogenic mutations leading to SVW cause loss of function of the protein encoded by the gene [16].

 Although we can estimate that the recurrence risk for future children of affected patients is 50%, it is still not possible to predict the severity of the disease in a fetus with a pathogenic mutation in *IRF6*, as there is no clear genotype-phenotype correlation. The pathogenic mutations in *IRF6* seem to play its major harmful effect during embryonic development, indicating that *IRF6* plays a critical functional role in craniofacial development. However,* IRF6* also seems to act after birth, as children with VWS have an increased frequency of wound complications after surgical cleft repair than children with NS CL ± P [18]. 

The spectrum of clinical variability of VWS has recently been expanded by the demonstration that mutations in *IRF6* are also causative of the Popliteal Pterygium Syndrome (PPS; OMIM 119500), an allelic, autosomal dominant disorder that presents, besides the facial anomalies typical of VWS, bilateral popliteal webs, syndactyly, and genital anomalies [17]. Most of the pathogenic mutations causative of PPS are located in exon 4 of the *IRF6* gene [16]. There are a strong genotype-phenotype correlation associated with VWS and PPS, but how the different mutations lead to PPS or VWS is still uncertain [19].

Since most of the VWS and PPS cases can be diagnosed by clinical evaluation, the necessity of genetic testing should be evaluated in each case. 

#### 2.1.2. Velocardiofacial Syndrome or 22q11.2 Deletion Syndrome

Velocardiofacial syndrome (VCFS; OMIM 192430) is an autosomal dominant disorder mainly characterized by the presence of cardiac anomalies (conotruncal defects, predominantly tetralogy of Fallot and conoventricular septal defects), CP or submucosal CP, velopharyngeal incompetence, facial dysmorphia, thymic hypoplasia, and learning disabilities [20]. The major known mutational mechanism causative of VCFS is a submicroscopic deletion at 22q11.2, usually spanning 1.5 Mb to 3 Mb. The spectrum of clinical variability is very wide, with the mildest cases presenting only two clinical signs of the syndrome in contrast to the full blown phenotype of the syndrome. Patients with DiGeorge syndrome (DGS; OMIM 188400), a condition with a great clinical overlap with VCFS, is also caused by deletions at 22q11.2, and thus represents a single entity; the term “22q11.2 deletion syndrome” is now commonly used to refer to all these cases. The clinical diagnosis for this group of patients is usually difficult, and genetic tests are often recommended in the presence of at least two clinical features of the syndrome, such as velopharingeal insufficiency and cardiac defects [21]. Moreover, patients may develop late onset psychosis or behavior disturbances, such as schizophrenia or bipolar disorders [22]. The severity of the syndrome is not dependent on the size of the deletion [23, 24] and several studies have pointed loss of one copy of *TBX1* as the major etiological agent within 22q11.2 leading to the phenotypic alterations [25, 26]. However, other environmental or genomic factors may also influence phenotype manifestation. Therefore, identification of 22q11.2 deletion patients is important for genetic counseling purposes as well as for discussing prognosis and surgical intervention, as the choice of surgical procedure depends upon the presence of abnormal and misplaced internal carotid arteries, which is relatively common in these patients (reviewed by Saman and Tatum [27]) The recurrence risk is high (50%) for carriers of the 22q11 deletion and it is still not possible to predict the severity of the disorder in fetuses with this alteration. 

#### 2.1.3. Robin Sequence and Associated Syndromes

Robin sequence (RS), also referred as Pierre Robin sequence, is characterized by the presence of micro or retrognathia, respiratory distress, and glossoptosis, with or without CP [28, 29]. It is also associated with high morbidity secondary to a compromised airway, feeding difficulties, and speech problems. It can occur isolatedly (called NS RS), but most of the time it is associated with a genetic syndrome [30]. Therefore, RS must not be regarded as a definitive diagnosis, and defining the presence of an associated syndrome has implications for future case management and determination of recurrence risks [30]. The most common syndromes associated with RS are Stickler syndrome and VCFS, both with an autosomal dominant pattern of inheritance and with several additional clinical complications that are not present in NS RS. 

The pathogenesis of NS RS is heterogeneous and not well defined. NS RS has been considered the result of intrauterine fetal constraint where extrinsic physical forces (e.g., oligohydramnios, breech position, or abnormal uterine anatomy) inhibit normal mandibular growth. Micrognathia in early fetal development may in turn cause the tongue to remain between the palatal shelves, thus interfering with palate closure [29, 31]. However, this mechanism has been challenged by the identification of several genetic alterations associated with RS, including chromosomal deletions such as 2q24.1-33.3, 4q32-qter, 11q21-23.1, and 17q21-24.3 [32] and microchromosomal deletions involving regulatory elements surrounding *SOX9* [33]. NS RS usually occurs as the unique case in the family and the recurrence risk for future pregnancies of the couple with one affected child is low [34].

### 2.2. Nonsyndromic CL ± P (NS CL ± P)

**Figure 1:**
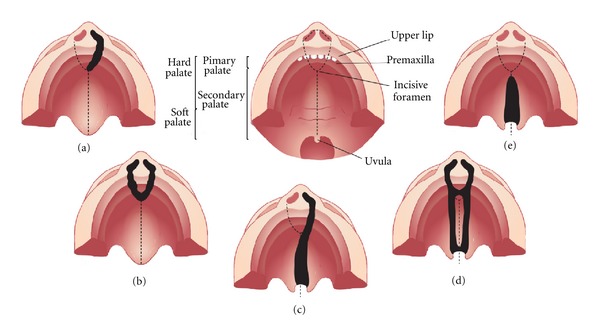
Representation of the most common types of cleft affecting the palate. (a) Unilateral cleft lip with alveolar involvement; (b) bilateral cleft lip with alveolar involvement; (c) unilateral cleft lip associated with cleft palate; (d) bilateral cleft lip and palate; (e) cleft palate only.

NS CL ± P includes a wide spectrum of clinical variability, from a simple unilateral lip scar to bilateral cleft lip and cleft of the palate, as partly represented in Figure 1. Different epidemiological evidence, as familial recurrence, observed in 20–30% of the cases [35, 36] and twin concordance rates (40–60% for monozygotic and 3–5% for dizygotic; [37]), suggest an important genetic component in NS CL ± P etiology. High heritability rates have been estimated in several studies (reaching 84% in Europe [38],78% in China [39] and 74% in South America [40]; in Brazil, our group found estimates ranging from 45% to as high as 85%, depending on the population ascertained [36]). The most accepted genetic model for NS CL ± P is the multifactorial, in which genetic and environmental factors play a role in phenotype determination.

Researchers have conducted different approaches to seek for genetic NS CL ± P susceptibility *loci*. Linkage analysis and association studiesof candidate genes were, initially, the most popular approaches, and the first gene suggested to be associated with NS CL ± P was transforming growth factor alpha (TGF*α*), by Ardinger et al. [41]. Thereafter, linkage analyses raised some other genomic regions as possible susceptibility factors, as 6p24-23 [42] (recently studied by Scapoli et al. [43]), 4q21 [44], 19q13 [45], and 13q33 [46]. Additional studies, however, faced a lack of reproducibility of the emerged genomic *loci*, as reviewed in detail by others [4, 47], suggesting the existence of a strong genetic heterogeneity underlying the predisposition to the disease (i.e., different causal *loci *might be acting in the different studied families). 

Candidate genes analyzed through association studies emerged not only from initial findings by linkage analysis, but also from: (1) the gene role in lip or palate embryogenesis, as suggested by animal model studies (e.g., *TGF*α**, in the pioneer study by Ardinger et al. [41] and* MSX1 *[48]); (2) gene role in the metabolism of putative environmental risk factors (e.g, *MTHFR*, involved in folate metabolism and firstly tested by Tolarova et al. [49], and *RAR*α*, *which encodes a nuclear retinoic acid receptor, tested initially by Chenevix-Trench et al. [50]); (3) from the identification of chromosomal anomalies in patients (as *SUMO1 *[51]), and (4) from their role in syndromic CL/P, such as van der Woude (*IRF6*, its causal gene, was firstly associated with NS CL ± P by Zucchero et al. [52]), Cleft Lip/Palate Ectodermal Dysplasia Syndrome (caused by mutations in *PVRL1 *[53], firstly associated with NS CL ± P by Sözen et al. [54]) and EEC and AEC (both caused by mutations in *TP63* [55], associated with NS CL ± P by Leoyklang et al. [56]), among others. 

 Among all *loci* that arose through linkage and candidate gene association studies, the *IRF6 *gene was the only *locus* to be consistently associated with NS CL ± P, as first shown by Zucchero et al. [52]. Rahimov et al. [57] identified a common nucleotide variant (namely rs642961) in an *IRF6 *regulatory sequence conferring risk to NS CL ± P that could potentially dysregulate *IRF6 *transcription levels and consequently dysregulate other signaling pathways. The variant rs642961 has been repeatedly associated in other studies in Europe [58, 59], Latin America [60, 61], and Asia, [62, 63]. Nevertheless, the role of rs641961 in embryonic development and how it predisposes to NS CL ± P remains to be elucidated.

**Figure 2:**
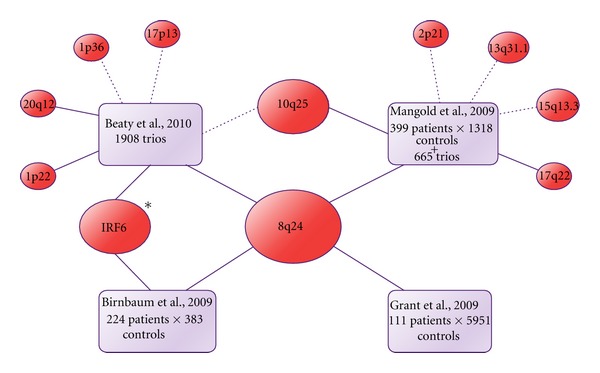
Diagram depicting the main lociassociated with NS CL ± P in the GWAS performed by Birnbaum et al. [72], Grant et al. [73], Mangold et al. [65], and Beaty et al. [64], which mixed case-control and trios (probands and their parents) approaches. Dotted lines represent borderline associations, whereas solid lines represent significant associations at the commonly accepted GWAS threshold (*P* < 10*E* − 7). (*) Mangold et al. [65] found evidence of interaction between IRF6 and GREM1, a gene located in 15q13.3 region, in NS CL ± P susceptibility.

With the advent of high-throughput genotyping technologies, which allowed for a deeper investigation at the genomic level without prior hypothesis of candidate regions to be tested, the landscape changed substantially. Genome-wide association studies (GWASs) came up from these advances, providing remarkable contribution to the understanding of NS CL ± P etiology. Four large GWASs were performed on NS CL ± P so far, and their main findings are summarized in Figure 2. Markers within a gene desert in the chromosomal region 8q24 were unequivocally implicated in NS CL ± P susceptibility, since they shared similar results. A second promising locus that emerged from these studies is the region 10q25. Other minor association studies have replicated association for both 8q24 and 10q25 [59, 60, 66–69]. Therefore, the *IRF6 *gene and the chromosomal regions 8q24 and 10q25 are, to date, the most corroborated *loci* implicated in NS CL ± P. However, contrary to *IRF6 *association, for which a punctual susceptibility variant has been identified, finding the functional causative mutations and the molecular pathogenesis beneath the associations observed for 8q24 and 10q25 regions remains a challenge; Table 1 summarizes the main candidate genes proposed by these studies. Recently, a GWAS performed in 34 consanguineous families from a Colombian isolated population suggested that the *loci *11p12, 11q25 and 8p23.2 may harbor recessive genes underlying NS CL ± P etiology [70]; these results, however, will need further replication. A recent linkage analysis applying high-throughput genotyping also suggested a role for the region of FOXE1 (9q22-q33) in NS CL ± P susceptibility [71]; nevertheless, this locus lacks reproducibility in other studies.

The difficulty of replication of the investigated *loci* may be a consequence of the genetic heterogeneity in NS CL ± P, that is, susceptibility variants differing from patient to patient; also, susceptibility variants may be different across unrelated populations. Beaty et al. [64] highlighted a stronger evidence for 8q24 in Europeans compared to Asians. Ethnic heterogeneity was also observed by Blanton et al. [67]; we have observed differences even across the Brazilian country populations [69], and a study with a Kenyan population failed in finding this association [74]. On the other hand, the Asians in the study reported by Beaty et al. [64] presented the most solid association for 20q12 and 1p22, compared to the European sample. It is possible that such differences may be a consequence of low statistical power in the subsample of a given ethnicity, as observed by Murray et al. [75]. Anyhow, these findings stress the value of testing non-European populations in order to identify the risk factors of NS clefting for each population, and to better understand the genetic architecture of the disease.

Regardless of the success of GWAS in identifying new susceptibility *loci*, those consistently implicated in NS CL ± P fail in explaining the complete genetic contribution proposed. This “failure” has been a common observation in many other traits, such as type 2 diabetes, height, and early onset myocardial infarction [76], and there is a current debate on where the remaining genetic causes could be hidden. One hypothesis is that gene-gene and gene-environment interactions may represent a substantial additional risk; however, their evaluation is still difficult with the current research tools. It is also possible that a combination of rare mutations per individual can be responsible for a large proportion of cases. New technologies to perform exome and genome sequencing are promising approaches to bridge this gap, and have potential to bring out new susceptibility variants. The use of other approaches, such as expression analysis, can also bring new insights into the causative pathways behind this malformation. In this respect, we have recently shown that dental pulp stem cells from NS CL ± P patients exhibit dysregulation of a set of genes involved in extracellular matrix remodeling, an important biological process for lip and palate morphogenesis [77]. 

### 2.3. Nonsyndromic CPO (NS CPO)

Cleft palate only is also a common malformation with a wide variability spectrum, comprising mildest phenotypes involving only uvula bifida to more severe cases, the majority of which include cleft of the soft and hard palates (Figure 1). The higher recurrence risk observed for close relatives compared to the general population [78, 79], and the higher concordance in monozygotic compared to dizygotic twins [80, 81] evidence the presence of genetic components in the etiology of NS CPO. Akin to NS CL ± P, NS CPO is believed to result from a combination of genetic and environmental factors [78]. However, in contrast to NS CL ± P, only a few studies on the genetic basis of NS CPO have been conducted, probably because of its lower prevalence and difficulty of ascertainment.

A first linkage genome scan to find NS CPO susceptibility loci was performed in 24 Finnish families by Koillinen et al. [82], which suggested 1p32, 2p24-25, and 12q21 as candidate regions; all of them, however, reached only borderline significance. Recently, Ghassibe-Sabbagh et al. [83] demonstrated the involvement of the Fas-associated factor-1 gene (*FAF1*) with NS CPO and provided insights into the gene's function in facial chondrogenic development, using a combination of an association study in a large multi-ethnic sample, gene expression analysis and animal model. Beaty et al., [84] performed a GWAS in 550 trios (proband and parents) of mixed ancestries and, although they did not find significant results by testing the associations of genetic markers with phenotype, they obtained interesting results when they performed the association tests conditioning on environmental variables (maternal smoking, alcohol consumption, and vitamin supplementation): association of TBK1, ZNF236, MLLT3, SMC2, and BAALC was suggested. None of the *loci* raised in these studies were in common with those emerged for NS CL ± P. Similarly, in search of a possible common etiology between NS CL ± P and NS CPO, many researchers tested the involvement of NS CL ± P candidate loci with NS CPO, but negative or conflicting results were reported for TGF*α*, TGF*β*3, MSX1, SUMO1, BCL3, IRF6 and 8q24 [57, 72, 85–90].

A number of studies in mice has shown that defects in several genes lead to cleft palate, often accompanied by a set of other defects, as reviewed by Cobourne [91]. Among those genes, the *MSX1 *was the most penetrant, that is, alterations in *MSX1 *led to CPO more frequently than alterations in other genes. Some authors have also reported chromosomal duplications, deletions and rearrangements in NS CPO patients [92–94]. Nonetheless, the genes located within those chromosomal regions lack confirmation with regards to their pathogenic role.

## 3. Genetic Management of the Family with CL/P-Affected Children

**Figure 3:**
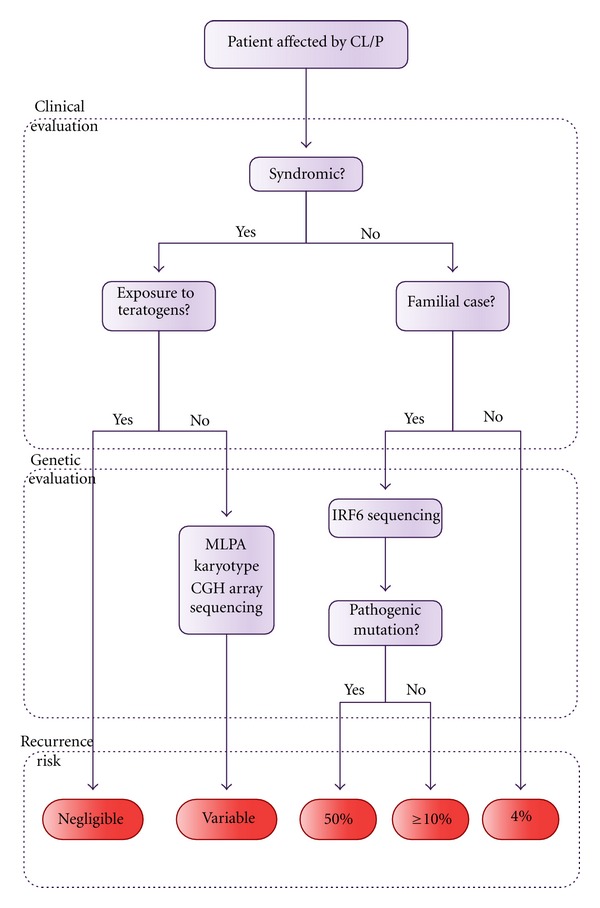
Flowchart depicting the genetic evaluation of a CL/P patient.

The clinical evaluation of a CL/P patient, outlined in Figure 3, starts with his/her classification in syndromic and nonsyndromic cases, based on the presence or absence of other dysmorphisms or malformations, together with an investigation of the occurrence of relatives with similar features.

Among the syndromic cases, it is first necessary to investigate the possibility of non-genetic causes, for example, exposure to teratogens during the first trimester of gestation. In cases of CL/P arising from the action of teratogenic agents during embryogenesis, the recurrence risk is negligible since exposure to teratogens in a next pregnancy does not recur. Once the possibility of a teratogenic origin for CL/P is ruled out, the geneticist should raise the diagnostic hypothesis of genetic syndromes and recommend the most adequate test (however, these tests might also be useful in the cases of teratogenic exposure, in order to refute chromosomal abnormalities). The most commonly performed tests are the karyotype, Multiplex Ligation-dependent Probe Amplification (MLPA), Comparative Genomic Hybridization array (CGH-array), gene target sequencing, and exome sequencing. Whilst the karyotype is a cytogenetic technique which allows for detection of large structural and numeric chromosomal anomalies in a low resolution, MLPA and CGH-array are quantitative molecular tests that enable the investigation of gain or loss of genetic material at the submicroscopic level. MLPA is applied to investigate specific targets in the genome while CGH-array can be used to screen the whole genome with a very high resolution. MLPA or CGH-array are the recommended tests to be used for a first screening, depending on the available resources [95, 96]. 

Gene target sequencing is recommended when one or more genes are known to be causative of the disorder. There is a trend towards the use of next generation sequencing particularly in diseases associated with genetic heterogeneity, as this approach permits the simultaneous testing of several genes, thus resulting in a more cost-effective test in the long run. Recurrence risk estimates for future children of the parents of one affected patient is dependent on the definition of the etiological mechanisms of the disease, evidencing the importance of selecting the appropriate test, combined with the clinical evaluation, for the establishment of the diagnosis.

In nonsyndromic cases, due to our full lack of understanding with regards to their etiological mechanisms, the recurrence risks have been empirically determined by epidemiological studies. As expected for a multifactorial model of inheritance, these risks can be influenced by several factors, such as gender of the affected propositus, severity of the orofacial cleft, and number of affected relatives [97]. The recurrence risk among families with one first-degree affected relative has been estimated as 4% for NS CL ± P and 2% for NS CPO [98]. These estimates may vary depending on the population. In Brazil, the recurrence risk has been estimated at only 2% among families with one first-degree NS CL ± P affected relative [36]. 

In NS cases, the identification of other individuals with CL/P in the family should be always interpreted with caution. Due to genetic heterogeneity associated with NS CL/P, a family with several affected individuals can actually represent the segregation of a single-gene disorder, which would not be promptly recognized based solely on clinical evaluation. For example, among 102 families with at least two individuals affected by NS CL/P, we identified 4 families with pathogenic mutations in *IRF6*, which actually represented VWS cases. Due to the high prevalence of VWS, we thus recommend *IRF6* genetic testing in familial cases of NS CL/P [99].

CL/P is a complex group of disorders and the adequate genetic management of the family requires evaluation by a trained group of geneticists in order to best define the diagnosis of the affected propositus, evaluation of prognosis, surgery indications, and, finally, recurrence risk estimates for the individuals at risk. With the advance of genomic technology, we expect that new advances and understanding of the genetic mechanisms leading to CL/P will be achieved in the upcoming years.

## Glossary

*Association Analysis:* correlates the occurrence, in two groups of individuals (e.g., affected and unaffected), of one genetic variant with the phenotype. If the frequency difference of one genotyped variant is statistically significant between the two groups, the genomic region harboring the variant will be associated with the trait. This approach is better suited to identify common and low impact genetic variants of shared origin.

*Exome Sequencing:* sequencing focused on the 2% of the genome which constitutes the protein-coding genes (exome). Despite the low proportion of the genome, 85% of the high-impact mutations already identified rely on the exome [100], which makes this approach highly promising. 

*Genetic Marker:* any polymorphism loci of known location which is suitable for gene mapping. Single nucleotide polymorphisms (SNPs), which involve one nucleotide substitution, are the most used for this purpose (e.g., in GWAS). A large number of SNPs can be analyzed simultaneously through the use of semi-automated equipments and microchips.

*GWAS:* association analysis at the genomic level. Requires the genotyping of thousands or millions of genetic markers, and has been made possible after advances in the characterization of the human genome (e.g., the Human Genome Project and the HapMap Project (http://www.hapmap.org/)) and automation of genotypic analysis. This strategy is suitable for identifying common low-effect variants without prior hypothesis. Finding association of the trait with a genetic marker does not necessarily mean that the marker is directly involved with the disease; most likely, the chromosomal region harboring this marker also comprises one or more susceptibility factors. Finding the real cause behind the association signal is currently a challenge. 

*Heritability:* fraction of phenotypic variance in a population attributable to genetic factors.

*Linkage Analysis:* approach that searches for genomic regions which cosegregate among affected individuals within a family, by genotyping known genetic markers spread throughout the genome. Powerful to detect genes of high impact, but loci of small or moderate effect are usually missed. Large families with many affected individuals are required. 

*Polymorphism:* genomic locus that admits two or more variants in the population and its rarest variant has a populational frequency greater than 1%.

*Whole-Genome Sequencing:* sequencing analysis of the whole genome, including coding and noncoding regions.

## Figures and Tables

**Table 1:** Main GWAS hits and genes possibly involved according to the authors.

Region	Possible gene involved	Function*
8q24	No know gene	
10q25	VAX1 [64]	Transcription factor, apparently involved in the development of the anterior ventral forebrain.
1p22	ABCA4 [64]	Transmembrane protein expressed in retinal photoreceptors. Mutations are involved with retinopathies.
17q22	NOG [65]	Secreted protein; binds and inactivates TGF*β* ^1^ proteins. Mutations are involved with bony fusion malformations, mainly in head and hands.
20q12	MAFB [64]	Transcription factor, acts in the differentiation and regulation of hematopoietic cell lineages. Mutations cause multicentric carpotarsal osteolysis syndrome.
1p36	PAX7 [64]	Transcription factor. Plays a role during neural crest development. Defects cause a form of rhabdomyosarcoma.
2p21	THADA [65]	Unclear function. Defects are related with thyroid tumors.
13q31.1	SPRY2 [65]	Citoplasm protein, colocalized with cytoskeleton proteins. Possibly acts as antagonist of FGF^2^.
15q13.1	FMN1 [65]	Peripheral membrane protein plays a role in cell-cell adhesion.
	GREM1 [65]	Secreted protein; BMP^3^ antagonist, expressed in fetal brain, small intestine, and colon.
17p13	NTN1 [64]	Extracellular matrix protein, mediates axon outgrowth and guidance. It may regulate diverse cancer tumorigenesis.

*According to OMIM database.

^1^Transforming growth factor beta.

^2^Fibroblast growth factor.

^3^Bone morphogenetic protein.

## References

[1] Mossey P, Castilla E Global registry and database on craniofacial anomalies.

[2] Mossey PA, Little J, Munger RG, Dixon MJ, Shaw WC (2009). Cleft lip and palate. *The Lancet*.

[3] Bender PL (2000). Genetics of cleft lip and palate. *Journal of Pediatric Nursing*.

[4] Dixon MJ, Marazita ML, Beaty TH, Murray JC (2011). Cleft lip and palate: understanding genetic and environmental influences. *Nature Reviews Genetics*.

[5] Kerrigan JJ, Mansell JP, Sengupta A, Brown N, Sandy JR (2000). Palatogenesis and potential mechanisms for clefting. *Journal of the Royal College of Surgeons of Edinburgh*.

[6] Jiang R, Bush JO, Lidral AC (2006). Development of the upper lip: morphogenetic and molecular mechanisms. *Developmental Dynamics*.

[7] Fraser FC (1974). Updating the genetics of cleft lip and palate. *Birth Defects*.

[8] Neilson DE, Brunger JW, Heeger S, Bamshad M, Robin E NH (2002). Mixed clefting type in Rapp-Hodgkin syndrome. *American Journal of Medical Genetics*.

[9] Kot M, Kruk-Jeromini J (2007). Analysis of family incidence of cleft lip and/or palate. *Medical Science Monitor*.

[10] Rutledge KD, Barger C, Grant JH, Robin NH (2010). IRF6 mutations in mixed isolated familial clefting. *American Journal of Medical Genetics A*.

[11] Stanier P, Moore GE (2004). Genetics of cleft lip and palate: syndromic genes contribute to the incidence of non-syndromic clefts. *Human Molecular Genetics*.

[12] Gabrielli S, Piva M, Ghi T (2009). Bilateral cleft lip and palate without premaxillary protrusion is associated with lethal aneuploidies. *Ultrasound in Obstetrics and Gynecology*.

[13] Schutte BC, Sander A, Malik M, Murray JC (1996). Refinement of the Van der Woude gene location and construction of a 3.5- Mb YAC contig and STS map spanning the critical region in 1q32-q41. *Genomics*.

[14] Burdick AB, Bixler D, Puckett Cl. (1985). Genetic analysis in families with Van Der Woude syndrome. *Journal of Craniofacial Genetics and Developmental Biology*.

[15] van der Woude A (1954). Fistula labii inferioris congenita and its association with cleft lip and palate. *The American Journal of Human Genetics*.

[16] Kondo S, Schutte BC, Richardson RJ (2002). Mutations in IRF6 cause Van der Woude and popliteal pterygium syndromes. *Nature Genetics*.

[17] Durda KM, Schutte BC, Murray JC IRF6-Related Disorders.

[18] Jones JLP, Canady JW, Brookes JT (2010). Wound complications after cleft repair in children with Van der Woude syndrome. *Journal of Craniofacial Surgery*.

[19] Little HJ, Rorick NK, Su LI (2009). Missense mutations that cause Van der Woude syndrome and popliteal pterygium syndrome affect the DNA-binding and transcriptional activation functions of IRF6. *Human Molecular Genetics*.

[20] Shprintzen RJ, Goldberg RB, Lewin ML (1978). A new syndrome involving cleft palate, cardiac anomalies, typical facies, and learning disabilities: velo-cardio-facial syndrome. *Cleft Palate Journal*.

[21] Sommerlad BC, Mehendale FV, Birch MJ, Sell D, Hattee C, Harland K (2002). Palate re-repair revisited. *Cleft Palate-Craniofacial Journal*.

[22] Shprintzen RJ, Goldberg R, Golding-Kushner KJ, Marion RW (1992). Late-onset psychosis in the velo-cardio-facial syndrome. *American Journal of Medical Genetics*.

[23] Carlson C, Sirotkin H, Pandita R (1997). Molecular definition of 22q11 deletions in 151 velo-cardio-facial syndrome patients. *American Journal of Human Genetics*.

[24] Sandrin-Garcia P, Abramides DVM, Martelli LR, Ramos ES, Richieri-Costa A, Passos GAS (2007). Typical phenotypic spectrum of velocardiofacial syndrome occurs independently of deletion size in chromosome 22q11.2. *Molecular and Cellular Biochemistry*.

[25] Chieffo C, Garvey N, Gong W (1997). Isolation and characterization of a gene from the DiGeorge chromosomal region homologous to the mouse Tbx1 gene. *Genomics*.

[26] Merscher S, Funke B, Epstein JA (2001). TBX1 is responsible for cardiovascular defects in velo-cardio-facial/DiGeorge syndrome. *Cell*.

[27] Saman M, Tatum SA (2012). Recent advances in surgical pharyngeal modification procedures for the treatment of velopharyngeal insufficiency in patients with cleft palate. *Archives of Facial Plastic Surgery*.

[28] Carey JC, Fineman RM, Ziter FM (1982). The Robin sequence as a consequence of malformation, dysplasia, and neuromuscular syndromes. *Journal of Pediatrics*.

[29] Evans KN, Sie KC, Hopper RA, Glass RP, Hing AV, Cunningham ML (2011). Robin sequence: from diagnosis to development of an effective management plan. *Pediatrics*.

[30] Shprintzen RJ (1992). The implications of the diagnosis of Robin sequence. *The Cleft Palate-Craniofacial Journal*.

[31] Schubert J, Jahn H, Berginski M (2005). Experimental aspects of the pathogenesis of Robin sequence. *Cleft Palate-Craniofacial Journal*.

[32] Jakobsen LP, Knudsen MA, Lespinasse J (2006). The genetic basis of the Pierre Robin Sequence. *Cleft Palate-Craniofacial Journal*.

[33] Benko S, Fantes JA, Amiel J (2009). Highly conserved non-coding elements on either side of SOX9 associated with Pierre Robin sequence. *Nature Genetics*.

[34] Sheffield LJ, Reiss JA, Strohm K, Gilding M (1987). A genetic follow-up study of 64 patients with the Pierre Robin complex. *American Journal of Medical Genetics*.

[35] Lie RT, Wilcox AJ, Skjaerven R (1994). A population-based study of the risk of recurrence of birth defects. *New England Journal of Medicine*.

[36] Brito LA, Cruz LA, Rocha KM (2011). Genetic contribution for non-syndromic cleft lip with or without cleft palate (NS CL/P) in different regions of Brazil and implications for association studies. *American Journal of Medical Genetics A*.

[37] Christensen K, Fogh-Andersen P (1993). Cleft lip (± cleft palate) in Danish twins, 1970–1990. *American Journal of Medical Genetics*.

[38] Calzolari E, Milan M, Cavazzuti GB (1988). Epidemiological and genetic study of 200 cases of oral cleft in the Emilia Romagna region of northern Italy. *Teratology*.

[39] Hu DN, Li JH, Chen HY (1982). Genetics of cleft lip and cleft palate in China. *American Journal of Human Genetics*.

[40] Menegotto BG, Salzano FM (1991). Clustering of malformations in the families of South American oral cleft neonates. *Journal of Medical Genetics*.

[41] Ardinger HH, Buetow KH, Bell GI, Bardach J, VanDemark DR, Murray JC (1989). Association of genetic variation of the transforming growth factor-alpha gene with cleft lip and palate. *American Journal of Human Genetics*.

[42] Eiberg H, Bixler D, Nielsen LS (1987). Suggestion of linkage of a major locus for nonsyndromic orofacial cleft with F13A and tentative assignment to chromosome 6. *Clinical Genetics*.

[43] Scapoli L, Martinelli M, Pezzetti F (2010). Expression and association data strongly support JARID2 involvement in nonsyndromic cleft lip with or without cleft palate. *Human Mutation*.

[44] Beiraghi S, Foroud T, Diouhy S (1994). Possible localization of a major gene for cleft lip and palate to 4q. *Clinical Genetics*.

[45] Stein J, Mulliken JB, Stal S (1995). Nonsyndromic cleft lip with or without cleft palate: evidence of linkage to BCL3 in 17 multigenerational families. *American Journal of Human Genetics*.

[46] Radhakrishna U, Ratnamala U, Gaines M (2006). Genomewide scan for nonsyndromic cleft lip and palate in multigenerational Indian families reveals significant evidence of linkage at 13q33.1-34. *American Journal of Human Genetics*.

[47] Carinci F, Scapoli L, Palmieri A, Zollino I, Pezzetti F (2007). Human genetic factors in nonsyndromic cleft lip and palate: an update. *International Journal of Pediatric Otorhinolaryngology*.

[48] Satokata I, Maas R (1994). Msx1 deficient mice exhibit cleft palate and abnormalities of craniofacial and tooth development. *Nature Genetics*.

[49] Tolarova M, van Rooij I, Pastor M (1998). A common mutation in the MTHFR gene is a risk factor for nonsyndromic cleft lip and palate anomalies. *The American Journal of Human Genetics*.

[50] Chenevix-Trench G, Jones K, Green AC, Duffy DL, Martin NG (1992). Cleft lip with or without cleft palate: associations with transforming growth factor alpha and retinoic acid receptor loci. *American Journal of Human Genetics*.

[51] Alkuraya FS, Saadi I, Lund JJ, Turbe-Doan A, Morton CC, Maas RL (2006). SUM01 haploinsufficiency leads to cleft lip and palate. *Science*.

[52] Zucchero TM, Cooper ME, Maher BS (2004). Interferon regulatory factor 6 (IRF6) gene variants and the risk of isolated cleft lip or palate. *New England Journal of Medicine*.

[53] Suzuki K, Hu D, Bustos T (2000). Mutations of PVRL1, encoding a cell-cell adhesion molecule/herpesvirus receptor, in cleft lip/palate-ectodermal dysplasia. *Nature Genetics*.

[54] Sözen MA, Suzuki K, Tolarova MM, Bustos T, Fernández Iglesias JE, Spritz RA (2001). Mutation of PVRL1 is associated with sporadic, non-syndromic cleft lip/palate in northern Venezuela. *Nature Genetics*.

[55] Rinne T, Brunner HG, van Bokhoven H (2007). p63-associated disorders. *Cell Cycle*.

[56] Leoyklang P, Siriwan P, Shotelersuk V (2006). A mutation of the p63 gene in non-syndromic cleft lip. *Journal of Medical Genetics*.

[57] Rahimov F, Marazita ML, Visel A (2008). Disruption of an AP-2*α* binding site in an IRF6 enhancer is associated with cleft lip. *Nature Genetics*.

[58] Birnbaum S, Ludwig KU, Reutter H (2009). IRF6 gene variants in Central European patients with non-syndromic cleft lip with or without cleft palate. *European Journal of Oral Sciences*.

[59] Mostowska A, Hozyasz KK, Wojcicki P, Biedziak B, Paradowska P, Jagodzinski PP (2010). Association between genetic variants of reported candidate genes or regions and risk of cleft lip with or without cleft palate in the polish population. *Birth Defects Research A *.

[60] Rojas-Martinez A, Reutter H, Chacon-Camacho O (2010). Genetic risk factors for nonsyndromic cleft lip with or without cleft palate in a mesoamerican population: evidence for IRF6 and variants at 8q24 and 10q25. *Birth Defects Research A*.

[61] Brito LA, Bassi C, Masotti C (2012). IRF6 is a risk factor for nonsyndromic cleft lip in the Brazilian population. *American Journal of Medical Genetics A*.

[62] Pan Y, Ma J, Zhang W (2010). IRF6 polymorphisms are associated with nonsyndromic orofacial clefts in a Chinese Han population. *American Journal of Medical Genetics A*.

[63] Shi J, Song T, Jiao X, Qin C, Zhou J (2011). Single-nucleotide polymorphisms (SNPs) of the IRF6 and TFAP2A in non-syndromic cleft lip with or without cleft palate (NSCLP) in a northern Chinese population. *Biochemical and Biophysical Research Communications*.

[64] Beaty TH, Murray JC, Marazita ML (2010). A genome-wide association study of cleft lip with and without cleft palate identifies risk variants near MAFB and ABCA4. *Nature Genetics*.

[65] Mangold E, Ludwig KU, Birnbaum S (2010). Genome-wide association study identifies two susceptibility loci for nonsyndromic cleft lip with or without cleft palate. *Nature Genetics*.

[66] Nikopensius T, Ambrozaityte L, Ludwig KU (2009). Replication of novel susceptibility locus for nonsyndromic cleft lip with or without cleft palate on chromosome 8q24 in Estonian and Lithuanian patients. *American Journal of Medical Genetics A*.

[67] Blanton SH, Burt A, Stal S, Mulliken JB, Garcia E, Hecht JT (2010). Family-based study shows heterogeneity of a susceptibility locus on chromosome 8q24 for nonsyndromic cleft lip and palate. *Birth Defects Research A*.

[68] Nikopensius T, Birnbaum S, Ludwig KU (2010). Susceptibility locus for non-syndromic cleft lip with or without cleft palate on chromosome 10q25 confers risk in Estonian patients. *European Journal of Oral Sciences*.

[69] Brito LA, Paranaiba LM, Bassi CF (2012). Region 8q24 is a susceptibility locus for nonsyndromic oral clefting in Brazil. *Birth Defects Research A*.

[70] Camargo M, Rivera D, Moreno L (2012). GWAS reveals new recessive loci associated with non-syndromic facial clefting. *European Journal of Medical Genetics*.

[71] Moreno LM, Mansilla MA, Bullard SA (2009). FOXE1 association with both isolated cleft lip with or without cleft palate, and isolated cleft palate. *Human Molecular Genetics*.

[72] Birnbaum S, Ludwig KU, Reutter H (2009). Key susceptibility locus for nonsyndromic cleft lip with or without cleft palate on chromosome 8q24. *Nature Genetics*.

[73] Grant SF, Wang K, Zhang H (2009). A genome-wide association study identifies a locus for nonsyndromic cleft lip with or without cleft palate on 8q24. *The Journal of pediatrics*.

[74] Weatherley-White RC, Ben S, Jin Y, Riccardi S, Arnold TD, Spritz RA (2011). Analysis of genomewide association signals for nonsyndromic cleft lip/palate in a Kenya African Cohort. *American Journal of Medical Genetics A*.

[75] Murray T, Taub MA, Ruczinski I (2012). Examining markers in 8q24 to explain differences in evidence for association with cleft lip with/without cleft palate between Asians and Europeans. *Genetic Epidemiology*.

[76] Manolio TA, Collins FS, Cox NJ (2009). Finding the missing heritability of complex diseases. *Nature*.

[77] Bueno DF, Sunaga DY, Kobayashi GS (2011). Human stem cell cultures from cleft lip/palate patients show enrichment of transcripts involved in extracellular matrix modeling by comparison to controls. *Stem Cell Reviews and Reports*.

[78] Christensen K, Mitchell LE (1996). Familial recurrence-pattern analysis of nonsyndromic isolated cleft palate—a Danish registry study. *American Journal of Human Genetics*.

[79] Sivertsen A, Wilcox AJ, Skjærven R (2008). Familial risk of oral clefts by morphological type and severity: population based cohort study of first degree relatives. *BMJ*.

[80] Nordström REA, Laatikainen T, Juvonen TO, Ranta RE (1996). Cleft-twin sets in Finland 1948–1987. *Cleft Palate-Craniofacial Journal*.

[81] Grosen D, Bille C, Petersen I (2011). Risk of oral clefts in twins. *Epidemiology*.

[82] Koillinen H, Lahermo P, Rautio J, Hukki J, Peyrard-Janvid M, Kere J (2005). A genome-wide scan of non-syndromic cleft palate only (CPO) in Finnish multiplex families. *Journal of Medical Genetics*.

[83] Ghassibe-Sabbagh M, Desmyter L, Langenberg T (2011). FAF1, a gene that is disrupted in cleft palate and has conserved function in Zebrafish. *American Journal of Human Genetics*.

[84] Beaty TH, Ruczinski I, Murray JC (2011). Evidence for gene-environment interaction in a genome wide study of nonsyndromic cleft palate. *Genetic Epidemiology*.

[85] Hwang SJ, Beaty TH, Panny SR (1995). Association study of transforming growth factor alpha (TGF*α*) TaqI polymorphism and oral clefts: Indication of gene-environment interaction in a population-based sample of infants with birth defects. *American Journal of Epidemiology*.

[86] Shaw GM, Wasserman CR, Lammer EJ (1996). Orofacial clefts, parental cigarette smoking, and transforming growth factor-alpha gene variants. *American Journal of Human Genetics*.

[87] Shiang R, Lidral AC, Ardinger HH (1993). Association of transforming growth-factor alpha gene polymorphisms with nonsyndromic cleft palate only (CPO). *American Journal of Human Genetics*.

[88] Lidral AC, Romitti PA, Basart AM (1998). Association of MSX1 and TGFB3 with nonsyndromic clefting in humans. *American Journal of Human Genetics*.

[89] Mitchell LE, Murray JC, O’Brien S, Christensen K (2001). Evaluation of two putative susceptibility loci for oral clefts in the Danish population. *American Journal of Epidemiology*.

[90] Hecht JT, Mulliken JB, Blanton SH (2002). Evidence for a cleft palate only locus on chromosome 4 near MSX1. *American Journal of Medical Genetics*.

[91] Cobourne MT (2004). The complex genetics of cleft lip and palate. *European Journal of Orthodontics*.

[92] Brewer C, Holloway S, Zawalnyski P, Schinzel A, FitzPatrick D (1999). A chromosomal duplication map of malformations: regions of suspected haplo- and triplolethality—and tolerance of segmental aneuploidy—in humans. *American Journal of Human Genetics*.

[93] Brewer CM, Leek JP, Green AJ (1999). A locus for isolated cleft palate, located human chromosome 2q32. *American Journal of Human Genetics*.

[94] Brewer C, Holloway S, Zawalnyski P, Schinzel A, Fitzpatrick D (1998). A chromosomal deletion map of human malformations. *American Journal of Human Genetics*.

[95] DeVries S, Gray JW, Pinkel D, Waldman FM, Sudar D (2001). Comparative genomic hybridization. *Current Protocols in Human Genetics*.

[96] Jehee FS, Takamori JT, Vasconcelos Medeiros PF (2011). Using a combination of MLPA kits to detect chromosomal imbalances in patients with multiple congenital anomalies and mental retardation is a valuable choice for developing countries. *European Journal of Medical Genetics*.

[97] Grosen D, Chevrier C, Skytthe A (2010). A cohort study of recurrence patterns among more than 54000 relatives of oral cleft cases in Denmark: support for the multifactorial threshold model of inheritance. *Journal of Medical Genetics*.

[98] Gorlin RJ, Cohen MM, Hennekam RCM (2001). *Syndromes of the Head and Neck*.

[99] Jehee FS, Burin BA, Rocha KM (2009). Novel mutations in IRF6 in nonsyndromic cleft lip with or without cleft palate: When should IRF6 mutational screening be done?. *American Journal of Medical Genetics A*.

[100] Majewski J, Schwartzentruber J, Lalonde E, Montpetit A, Jabado N (2011). What can exome sequencing do for you?. *Journal of Medical Genetics*.

